# Flame-Retardant Properties and Mechanism of Polylactic Acid-Conjugated Flame-Retardant Composites

**DOI:** 10.3389/fchem.2022.894112

**Published:** 2022-05-11

**Authors:** Daohai Zhang, Meng Pei, Ke Wei, Fang Tan, Chengtao Gao, Dongmei Bao, Shuhao Qin

**Affiliations:** ^1^ School of Chemical Engineering of Guizhou Minzu University, Guizhou, China; ^2^ National Engineering Research Center for Compounding and Modification of Polymer Materials, Guizhou, China; ^3^ Polymer Composites Engineering Research Center of Guizhou Minzu University, Guiyang, China

**Keywords:** conjugated flame retardant, polylactic acid, flame retardant performance, gas phase flame retardant, composites

## Abstract

The DOPO derivative-conjugated flame retardant 4, 4'-{1'', 4'' - phenylene - bis [amino - (10‴ - oxy -10‴-hydro-9‴-hydrogen-10‴ λ^5^ -phosphaphenanthrene-10''-yl)-methyl]}-diphenol (P-PPD-Ph) with two hydroxyl groups was synthesized. Polylactic acid conjugated flame-retardant composites with P-PPD-Ph were papered by using a twin-screw extruder. The flame-retardant properties of polylactic acid-conjugated flame-retardant composites were investigated. The flame-retardant properties of PLA-conjugated flame-retardant composites were characterized by the limiting oxygen index (LOI) and the vertical burning test (UL94). The results showed that the PLA-conjugated flame-retardant composites achieved a V-0 rating (UL-94, 3.2 mm) when the conjugated flame retardant was added at 5 wt%, and increase in LOI value from 22.5% to 31.4% relative to composites without added conjugated flame retardant. The flame-retardant mechanism of PLA-conjugated flame-retardant composites were further studied by TG-FTIR, the results showed that the P-PPD-Ph promoted the PLA-conjugated flame-retardant composites to decompose and also released fragments with quenching and dilution, which suggests that P-PPD-Ph for PLA-conjugated flame-retardant composites mainly play a role of the gas-phase flame retardant.

## Introduction

In recent years, due to increasing depletion of petroleum resources and the pollution of petroleum-based polymers to the environment, environmentally friendly biodegradable materials have been rapidly developed (Bandehali et al., 2021). Among them, polylactic acid (PLA) receives more attention due to its excellent mechanical properties (Mirkhalaf and Fagerström, 2021), and it has been widely used in electrical and automotive applications as a replacement for some general-purpose plastics (Arroyo and Ryan, 2018; Li et al., 2018; Yang et al., 2022). However, PLA is an extremely flammable substance that will drip during the combustion process, thus limiting its application in the electrical industry (Sun et al., 2018; Ma et al., 2022). Therefore, the research on flame-retardant PLA is necessary. The addition of flame retardants is a common method for improving the flame retardancy of PLA, and there are many types of flame retardants suitable for PLA (Tawiah et al., 2018). Among them, organophosphorus compounds and their derivatives have received extensive attention because of their excellent flame retardancy and non-toxicity (Hirsch et al., 2017; Salmeia et al., 2018; Nazir and Gaan, 2020), and their applications are very broad (Wu et al., 2018; Bascucci et al., 2022; Wang et al., 2022).

In this series, 9,10-dihydro-9-oxa-10- phosphaphenanthrene-10-oxide (DOPO) and its derivatives have been hot in recent years. Schartel et al (2016) explored the flame retardant modes of action of DOPO derivatives, and DOPO derivatives have higher flame-retardant efficiency. Generally, there are two kinds of DOPO derivatives used to improve the flame-retardant properties of PLA. First, the additive DOPO derivatives are mixed into the polymer material, second, the reactive DOPO derivatives are directly reacted into the polymer chain. For instance, Long et al (2017) synthesized three kinds of Di-DOPO structures, namely, ethyl-DOPO, phenethyl-DOPO, and naphthalene-DOPO, and compounded them into PLA materials. Three kinds of PLA flame-retardant composites have good flame retardant performance, and at 10 wt% addition of the derivatives, all PLA flame-retardant composites achieved a V-0 rating (UL-94, 3.2 mm). As additive flame retardants, DOPO derivatives have shown good flame-retardant ability for polymers. However, additive DOPO derivatives exhibit practical drawbacks during application, such as migration problems and the deterioration of polymer performance (Chu et al., 2022). Therefore, introducing in active groups that react with the matrix in DOPO derivatives is a current essential work. Researchers have tried to introduce a variety of DOPO derivatives with different reactive groups. For instance, Tao et al. (Yu et al., 2017) synthesized of the 9,10-dihydro-9-oxa-10-phosphaphenanthrene-10-oxide and 1,6-hexane diisocyanate (DOPO-ICN) and applied it to jute/PLA flame-retardant composites. DOPO-ICN reacts with PLA, and this makes DOPO-ICN to not only improve the flame retardant properties of PLA flame-retardant composites but also create less impact on other properties of composites.

**FIGURE 1:**
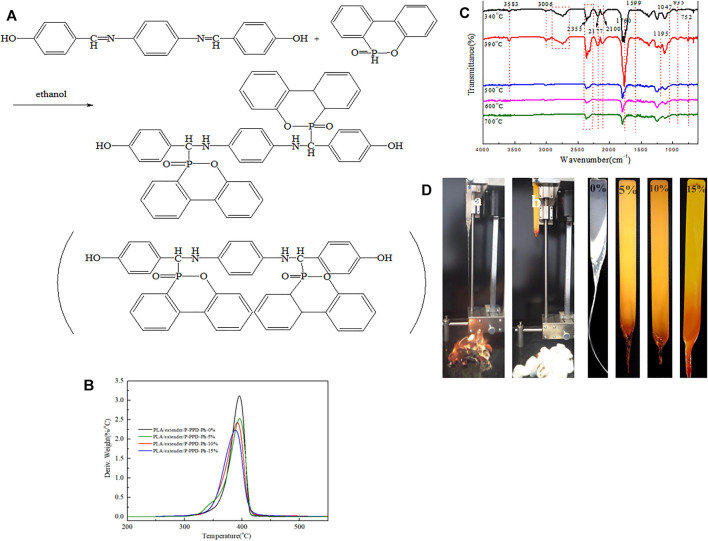
**(A)** Schematic diagram of synthetic P-PPD-Ph. **(B)** Thermal analysis of PLA and PLA-conjugated flame-retardant composites under nitrogen (N_2_) atmosphere. **(C)** FTIR spectra of the PLA-conjugated flame-retardant composites at different temperatures of TG under N_2_ atmosphere. **(D)** Photos of samples during the UL-94 test (a is PLA/P-PPD-Ph-0%, and b is PLA/P-PPD-Ph-5%).

In this study, a reactive DOPO derivative (P-PPD-Ph conjugated flame retardant) was synthesized (as Figure 1A shows) and added the P-PPD-Ph into the PLA matrix. The flame retardant properties of PLA-conjugated flame retardant composites were characterized by limiting oxygen index (LOI) and vertical combustion test (UL94).

## Materials and Methods

### Materials

9,10-Dihydro-9-oxa-10-phosphaphenanthrene10-oxide (DOPO) was purchased from Huawei Ruike Chemical Co., Ltd. (Beijing, China). PLA was purchased from Wuxi Diaisheng Epoxy Co., Ltd. (Wuxi, China). Epoxy chain extender (ADR-5481) was purchased from Sinopharm Group Chemical Reagent Co., Ltd. (Shanghai, China).

### Preparation of PLA-Conjugated Flame-Retardant Composites

Imine-containing compounds and P-PPD-Ph were synthesized according to literature procedures (Sun and Yao, 2011). PLA, chain extender, and P-PPD-Ph were dried for 4 h under vacuum at 80°C before use. PLA, epoxy chain extender (4 wt‰), and P-PPD-Ph (0, 5, 10, and 15 wt%) were mixed uniformly. Then, the mixture was extruded by using a twin-screw extruder (CTE 35, Coperion Keya Machinery Manufacturing Co., Ltd., China) at a temperature of 180–200°C and a screw speed of 300 rpm. The extruded pellets were then molded into samples for testing using an injection molding machine (CJ80MZ 2N CII, Zhende Plastic Machinery Factory, China) at 180–200°C.

### Characterization

Thermal analysis (TG) was conducted in a Q50 thermal gravimetric analyzer (TA, United States) at a heating rate of 10°C/min and under nitrogen conditions. Approximately, 5 mg of the sample was weighed and placed in an aluminum ceramic with a gas flow of 60 ml/min.

The UL-94 test (SH5300, Guangzhou Xinhe Electronic Equipment Co., Ltd., China) was conducted with sample sizes of 130*10*3.2 mm^3^ in accordance with the ASTM UL 94-2006.

The LOI measurements were performed on an oxygen index flammability gauge (HC-2C) according to ASTM D 2863-97 standard with a sample dimension of 100 *6.5 *3.2 mm^3^.

## Results and Discussion

### Thermal Degradation Behavior of PLA-Conjugated Flame-Retardant Composites

The TG curves under N_2_ of PLA and PLA-conjugated flame-retardant composites containing P-PPD-Ph are presented in Figure 1B, and the corresponding data are listed in Table 1A. Relative to PLA/materials without the addition of flame retardant, the addition of flame-retardant P-PPD-Ph reduced the initial decomposition temperature (T−_5%_) of PLA-conjugated flame-retardant composites. This may be due to the decomposition temperature of P-PPD-Ph before PLA, and the decomposition of P-PPD-Ph changed the decomposition behavior and induced the PLA-conjugated flame-retardant composites to decompose in advance. Additionally, the residual carbon of PLA-conjugated flame- retardant composites at 700°C increased with the addition of the P-PPD-Ph conjugated flame retardant. This is due to the degradation of phosphorus-containing groups generating heat-resistant residues, which lead to inhibit the decomposition of PLA-conjugated flame-retardant composites (Battig et al., 20121), reduce the maximum thermal decomposition rate, and promote the formation of carbon layers, thereby increasing the char yield of PLA-conjugated flame-retardant composites. Also, when the addition of P-PPD-Ph conjugated flame retardant was 15 wt%, the char residue of the PLA-conjugated flame-retardant composites increased by 4.2% under 700°C. This shows that P-PPD-Ph conjugated flame retardant creates a condensed-phase flame-retardant effect on PLA-conjugated flame-retardant composites.

**TABLE 1A:** (A) TG and DTG data of PLA and PLA-conjugated flame-retardant composites derived from TG analysis.

Sample	T_5_% (°C)	T_max_ (°C)	Value at T_max_ (%/°c)	Residue
PLA/P-PPD-Ph-0%	344	395	3.1	0
PLA/P-PPD-Ph-5%	342	395	2.5	3.6
PLA/P-PPD-Ph-10%	345	392	2.4	3.2
PLA/P-PPD-Ph-15%	340	388	2.2	4.2

### The TG-FTIR of PLA-Conjugated Flame-Retardant Composites

Figure 1C shows the FTIR spectra of composites at different temperatures in the TG test under N_2_ conditions. In the PLA/P-PPD-Ph-15% initial decomposition stage (around 340°C), the absorption peak of P-O near 935 cm^−1^ appeared in the FTIR spectrum, and 1,599 cm^−1^ was the absorption peak of P-Ph, and the absorption peaks of CO_2_ (2,355 cm^−1^) and CO (2,177 cm^−1^ and 2,100 cm^−1^) appeared. The stretching vibration peak of carbonyl was near 1760 cm^−1^, and the absorption peak of C–H on the aliphatic chain was at 3,006 cm^−1^. Also, the absorption peak of hydrocarbons and carbon–oxygen species was 2,638–2,908 cm^−1^, and the absorption peak of -OH was 3,583 cm^−1^. With the increase of temperature to the maximum decomposition temperature (about 390°C), the thermal decomposition products of PLA/P-PPD-Ph-conjugated flame-retardant composites were detected by FTIR, and the absorption peak intensity was the strongest and new absorption peaks were detected. 752cm^−1^ may be the absorption peak of P-C, 1195cm^−1^ may be the absorption peak of P=O, and 935, 1,047, and 1195cm^−1^ were the absorption peaks of aromatic phosphorus-containing complexes, which was consistent with the related literature (Yu et al., 2020; Qin et al., 2021). It showed that the thermal decomposition of the PLA conjugated flame-retardant composites may release aromatic esters and aromatic phosphorus-containing compounds. These substances were beneficial to improve the flame retardancy of the conjugated flame-retardant composites and played a gas-phase flame-retardant effect. However, as the thermal decomposition temperature increased to 500°C, the peak intensity of FTIR significantly decreased, and the absorption peaks at 3,006 cm^−1^ (C–H) and 2,638–2,908 cm^−1^ (hydrocarbon and carbon–oxygen species, respectively) disappeared. When the temperature reached 600°C (final decomposition stage), the absorption peaks of P=O at 1195cm^−1^ and 3583cm^−1^ (-OH) disappeared, which corroborated that the thermal decomposition products of the conjugated flame-retardant composites may have PO_2_· and PO·, which can capture H· and OH·, so P-PPD-Ph played a gas-phase flame-retardant role in PLA-conjugated flame-retardant composites.

### Effects of the P-PPD-Ph on the Flame-Retardant Properties of PLA-Conjugated Flame-Retardant Composites

The effects of the P-PPD-Ph conjugated flame retardant on the flame retardant performance of PLA-conjugated flame-retardant composites were investigated by the vertical burning test (UL-94) and limiting oxygen index test (LOI), and the results are shown in Table 1B. As shown in Table 1B, when the content of P-PPD-Ph conjugated flame retardant is 5 wt%, the PLA-conjugated flame-retardant composites can pass the UL-94 V-0 level. As shown in Figure 1D, the melting dripping occurred in PLA and also in the PLA-conjugated flame-retardant composites. In PLA materials without the addition of flame retardant, splines were dropped in the form of wire-drawing after combustion, while after the P-PPD-Ph conjugated flame retardant was added, the molten droplets after combustion were dropped in the form of water drops. Moreover, PLA without the addition had droplets with fire, and the absorbent cotton was easily ignited, whereas the PLA conjugated flame retardant composites with 5% P-PPD-Ph added had droplets more rapidly without fire, and the absorbent cotton was well reserved. The results show that P-PPD-Ph can decompose the matrix in advance, thus producing molten droplets that take away the heat of the burning matrix (Xi et al., 2016), which is the same as the explanation on TG-FTIR of composites; the quenching and dilution effects of P-PPD-Ph can extinguish the fire on droplets during dripping, thereby the cotton will not be ignited. Also, the LOI value of PLA conjugated flame retardant composites increased from 22.5 to 34.0%, it achieved a good flame-retardant effect, and it can be seen that the prepared P-PPD-Ph conjugated flame retardant had higher flame-retardant efficiency.

**TABLE 1B:** (B) UL-94 and LOI test results for PLA-conjugated flame-retardant composites.

Sample	LOI (%)	UL-94 (3.2 mm)			
		t_1_/t_2_ (s)	Dripping	Ignition	Rating
PLA/P-PPD-Ph-0%	22.5	BC	Yes	Yes	—
PLA/P-PPD-Ph-5%	31.4	3.45/0.23	Yes	No	V-0
PLA/P-PPD-Ph-10%	33.6	1.37/0.17	Yes	No	V-0
PLA/P-PPD-Ph-15%	34.0	0.37/0.18	Yes	No	V-0

## Conclusion

In this study, P-PPD-Ph conjugated flame retardant with a double hydroxyl DOPO derivative was synthesized and reacted with the PLA matrix to prepare PLA-conjugated flame-retardant composites. The effects of P-PPD-Ph conjugated flame retardant content on the flame retardant performance of PLA composites were investigated. When the content of P-PPD-Ph conjugated flame retardant was 5 wt%, the PLA conjugated flame retardant composites passed the V0 grade (UL-94). Therefore, the P-PPD-Ph conjugated flame retardant has an effective flame-retardant effect on PLA. TG-FTIR tests showed that the compound has the inhibitory flame mechanism of the gas phase and condensed phase in PLA and has a good effect on promoting the formation of PLA-conjugated flame-retardant composites into carbon. When the V0 level is reached, the PLA-conjugated flame-retardant composites still have good mechanical properties.

## Data Availability

The raw data supporting the conclusion of this article will be made available by the authors, without undue reservation.
